# Assessment of the effectiveness of BOPPPS-based hybrid teaching model in physiology education

**DOI:** 10.1186/s12909-022-03269-y

**Published:** 2022-03-30

**Authors:** Xiao-Yu Liu, Chunmei Lu, Hui Zhu, Xiaoran Wang, Shuwei Jia, Ying Zhang, Haixia Wen, Yu-Feng Wang

**Affiliations:** Department of Physiology, School of Basic Medical Sciences, Harbin Medical University, 157 Baojian Rd, Nangang, Harbin, 150081 Heilongjiang China

**Keywords:** BOPPPS, Hybrid teaching, Online teaching, Physiology education, Teaching model

## Abstract

**Background:**

Online teaching has become increasingly common in higher education of the post-pandemic era. While a traditional face-to-face lecture or offline teaching remains very important and necessary for students to learn the medical knowledge systematically, guided by the BOPPPS teaching model, combination of online and offline learning approaches has become an unavoidable trend for maximizing teaching efficiency. However, in physiological education, the effectiveness of combined online teaching and offline teaching models remains poorly assessed. The present study aims at providing an assessment to the hybrid teaching model.

**Methods:**

The study was performed among undergraduate medical students of Class 2017 ~ 2019 in the Physiology course in Harbin Medical University during 2018-2020. Based on established offline teaching model with BOPPPS components in 2018, we incorporated online teaching contents into it to form a hybrid BOPPPS teaching model (HBOPPPS, in brief), preliminarily in 2019 and completely in 2020. HBOPPPS effectiveness was assessed through comparing the final examination scores of both objective (multi-choice and single answer questions) and subjective (short and long essays) questions between classes taught with different modalities.

**Results:**

The final examination score of students in Class 2019 (83.9 ± 0.5) who were taught with the HBOPPPS was significantly higher than that in Class 2017 (81.1 ± 0.6) taught with offline BOPPPS and in Class 2018 (82.0 ± 0.5) taught with immature HBOPPPS. The difference mainly attributed to the increase in average subjective scores (41.6 ± 0.3 in Class 2019, 41.4 ± 0.3 in Class 2018, and 38.2 ± 0.4 in Class 2017). In the questionnaire about the HBOPPPS among students in Class 2019, 86.2% responded positively and 79.4% perceived improvement in their learning ability. In addition, 73.5% of the students appreciated the reproducibility of learning content and 54.2% valued the flexibility of HBOPPPS. Lastly, 61.7% of the students preferred the HBOPPPS relative to BOPPPS in future learning.

**Conclusions:**

HBOPPPS is likely a more effective teaching model and useful for enhancing effectiveness of Physiology teaching. This is attributable to the reproducibility and flexibility as well as the increased learning initiatives.

**Supplementary Information:**

The online version contains supplementary material available at 10.1186/s12909-022-03269-y.

## Background

With the development of science and technology, web-based E-learning has been applied extensively in modern medical education, which reached a peak in 2020 when COVID-19 pandemic caused suspension of offline teaching in all educational institutions [1, 2]. Classically, E-learning like video lectures [3–5] mainly serves as supplements for offline lecture and is less effective in resolving individuals’ specific problems, inspiring students’ enthusiasm and matching the course objectives. During the COVID-19 pandemic, most of online teaching duplicates the methods of offline teaching by introducing the functions of screen share, checking attendance, “raising hands” and “quizzes”, and so on [6]. The online teaching is more effective in spatiotemporal efficiency [7–9]; however, its disadvantages are also obvious, such as low enthusiasm of students’ participation and insufficient network connectivity [8, 9]. Thus, a hybrid teaching method that combines online and offline teaching advantages is highly demanded in the post-pandemic era [10–12].

In modern medical education, offline teaching is commonly guided by a BOPPPS teaching theory that is the abbreviation of Bridge-in, Objectives and Outcomes of Learning, Pre-assessment, Participatory learning, Post-assessment and Summary. It was first introduced by Douglas Kerr from the University of British Columbia, and has been developed recently in medical education in China [13–15]. The BOPPPS is generally helpful for teachers to organize teaching process clearly and inspires students’ initiatives to learn [13]. However, the time limitation of offline teaching restrains its applicability, particularly the methods of pre- and post-assessments, and summary. In addition, it is relatively weak in enriching students’ knowledge, establishing feed-forward and feedback connections between students and teachers, targeting general obstacles and meeting diverse learning style preferences among medical students [16]. Thus, incorporating online teaching into the BOPPPS and optimizing its individual components should be considered.

To resolve this issue, in teaching Physiology for medical students in Harbin Medical University, we designed a new hybrid teaching model, i.e. HBOPPPS that incorporates online teaching into a new BOPPPS teaching model based on our understandings of learning characteristics of medical students [17]. We selected Physiology because it is an important discipline in medical education and the bridge between basic medical science and clinical medicine while efficiency of hybrid teaching model in Physiology education has not been fully assessed. This HBOPPPS model was optimized with some components of TESOT that is a teaching modality enriched with components of student-centered approach and online interactions targeting the learning obstacles to medical students worldwide [16]. We first performed the hybrid teaching on Physiology in the fall of 2019 for the students of Class 2018, and then in 2020 for Class 2019. The efficiency of this new model was evaluated by the final examination scores which were compared to that from Class 2017 taught with conventional BOPPPS only in 2018. A questionnaire was then performed among students of Class 2019 to investigate students’ learning abilities and their assessments of the HBOPPPS.

## Methods

### Study subjects

This study included 1576 students of clinical medicine major from 3 academic calendar years, i.e., 532 from Class 2017, 526 from Class 2018 and 518 from Class 2019 in Harbin Medical University in China. The students in the 3 years were from almost all regions of China and approximate half of them were locals of Heilongjiang province (48.0, 53.0 and 53.0% of Class 2017, 2018 and 2019, respectively). They all received systematic pre-college education under the same guideline and using the same textbooks while passing the requirements of entrance examination. The students in each year were divided into 5 sections, ~ 100 students/section, to be taught separately. Each section was usually taught by six teachers throughout the whole Physiology course of 80 sessions with 45 min/session in the second school year. Each teacher was responsible for 2 of 12 chapters (Supplemental Table 1). Because of the huge number of students and multiple sections at the same time period, teachers were not exactly the same in the 3 years or even in the same year. However, all teachers had at least 5 years’ experience of teaching and met the standard requirements of lecturing after group rehearsal of the course contents. These practices made the courses given by different teachers comparable.

Class 2017 was used as the control group of traditional BOPPPS teaching model (Table 1). In applying the HBOPPPS, we first incorporated online teaching method into BOPPPS teaching for the students of Class 2018 in the fall of 2019, mainly by posting micro-video lectures online without considering TESOT components. We further improved the HBOPPPS teaching model in teaching Class 2019 by including online guidance of the ongoing lectures and by adding a pre-lecture quiz at the beginning and post-lecture quizzes and students-led summary at the end of the lecture used in TESOT [16] as the final model of HBOPPPS.

**Table 1:** Constitution of BOPPPS

BOPPPS	Mode
**B**ridge-in	A course contents-associated video or image was presented to attract students’ attention
**O**bjective/**O**utcomes	What the students will gain from the lecture, i.e., what students are able to do or know by the end of the lecture.
**P**re- assessment	Single/multiple choice questions or topics for discussion at the first 5 min in the lecture
**P**articipatory learning	Group discussions, student presentations
**P**ost-assessment	Single/multiple choice questions, discussions
**S**ummary	Synopsis explained by teachers

### HBOPPPS

**Fig. 1:**
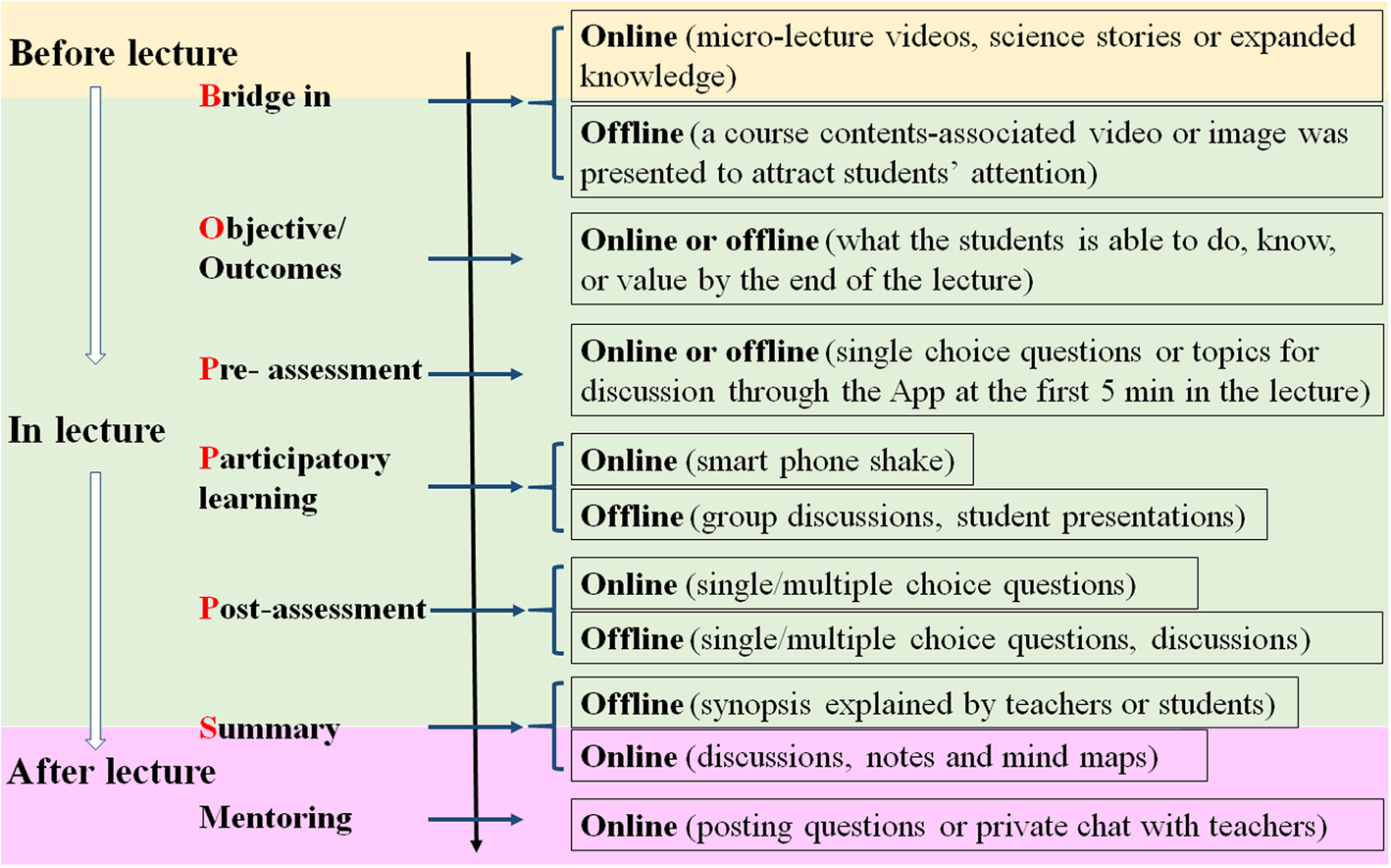
Constitution design of HBOPPPS

HBOPPPS was designed by incorporating online teaching into the enriched BOPPPS to form a hybrid teaching method. HBOPPPS model was applied through the following three stages (Fig. 1 and Supplemental Table 2).

1) Before the lecture. Teachers posted guidance of the course through a Mobile App called “Xuexi Tong” installed in Smartphone, which included the teaching schedule, introduction of course objectives, the key knowledge to be mastered, goal of ability nurturing (i.e. summarizing the course contents) and emotional attitude and values (i.e. understanding the position of undergoing course in whole medical system, developing good learning habits, and finding effective learning methods). These contents aimed at informing the students what the learning goals of the course are and how they could achieve the goals. In the Mobile App, course-associated micro-lecture videos (80 in total, 5 ~ 10 min/video, 1 ~ 2 videos/session) based on a text book of Physiology (Ting-Huai Wang, People’s Medical Publishing House, 9th Ed) were posted online to inform the students about the outline teaching of the ongoing course and key contents, at least 1 day before the lecture. Students were required to watch them completely before the lecture. Some opening questions about the ongoing lecture were also posted to guide students to get a preliminary answer through searching textbooks or online resources by themselves. In addition, some course-associated science stories and clinical cases were posted to expand students’ knowledge and enforce clinical association of the ongoing course contents.

2) In the lecture. After a sign-in through the App at 10 min before the lecture in classroom, some course-relevant topics/events were given to attract students’ attention into the course, such as mentioning respiratory symptoms of COVID-19 patients before introducing respiratory functions, or broadcasting a heavy breathing sound to make students curious about the ongoing course contents, or showing an image of the histology of the respiratory system to remind of students of previously learned knowledge. An earlier posted micro-lecture video or other associated short teaching video was played to generate quizzes from simple factual contents to analytic consideration to inspire students’ thinking. Alternatively, a single/multiple choice question (Supplemental Table 3) was sent to the students as a pre-assessment via the Mobile App. The answers of students to the questions could be calculated automatically by the App, which generated a statistical graph of the answers and gave a direct reference for the teacher to catch the major problems that likely puzzled the students. This information allowed the teacher to adjust main lecture contents and explain why the right answer should be selected and how the wrong answers should be avoided in the subsequent main lectures.

In the main lecture, to enhance students’ initiatives in learning, several interactive sessions were carried out, e.g. group discussion, student-led presentation of specific contents and clinical case analysis. A smart phone shake was used to select student(s) to answer questions randomly in these processes. These activities were designed to enhance students’ interest in learning and deepen their understanding and memory of course contents.

After the “participatory learning”, a post-assessment was performed with 3 ~ 5 multi-choice questions to evaluate how well the students mastered the knowledge and whether the aims of course were achieved. This assessment also gave teacher the opportunity to help students correct their misunderstandings of the course contents through summarizing the course contents, online discussion or feedback at the beginning of next lecture. At last, a summary of the course contents was given by the teacher or the students under the supervision of the teacher to highlight the key contents based on blackboard writing with emphasis marks and associated figures in PPT.

3) After the lecture: A mind map (Supplemental Fig. 1) was posted online by the teacher to guide students to summarize the lecture contents by themselves. All students posted their course summary online for further discussion, which aimed at helping students build a holistic view of the course. The students could ask questions individually about the course and the mind map through internet by posting questions in the discussion section in the Mobile App or having a private conversation with teachers, which let them get the answers without limitation of the time and space.

### Evaluation

To assess the teaching effectiveness of applying HBOPPPS model, we compared the final scores between different teaching model groups. The final examination of Physiology course was worthy of 100 points, consisting of 50 multi-choice and single answer questions (1 point/question) and 50 points of subjective questions, i.e., 6 short (5 points each) and 2 long (10 points each) essays, example of which is shown in Supplemental Table 4. The contents of the final exam in the 3 years were different from each other, randomly drawn from a test bank. However, they had the same score distribution for different chapters with similar difficulty index. In addition, there was also an online score generated automatically by the system of the Mobile App, which was used to assess students’ initiatives of self-learning. This online score consisted of micro-lecture video watching (30 points), homework for each chapter (40 points), test (10 points) and the attendance (20 points).

Lastly, we did a survey through questionnaires (Supplemental Table 5) in Class 2019 at the end of the semester of 2020 to analyze the effect of HBOPPPS on students’ learning ability, which included finding, analyzing and solving a problem and teamwork. A total of 506 valid questionnaires were collected and analyzed.

### Data analysis

All analysis was performed using SigmaStat program (SPSS 19, Chicago, IL). Comparisons of the final scores between different years were performed using one-way ANOVA followed by Bonferroni or Dunnett T3 test. Correlation analysis was performed between individuals’ final score and the online score. Comparisons between rates were carried out using chi-square test. Data are expressed as mean ± SEM and *P* < 0.05 was considered statistically significant.

## Results

### Students’ final examination scores

**Fig. 2:**
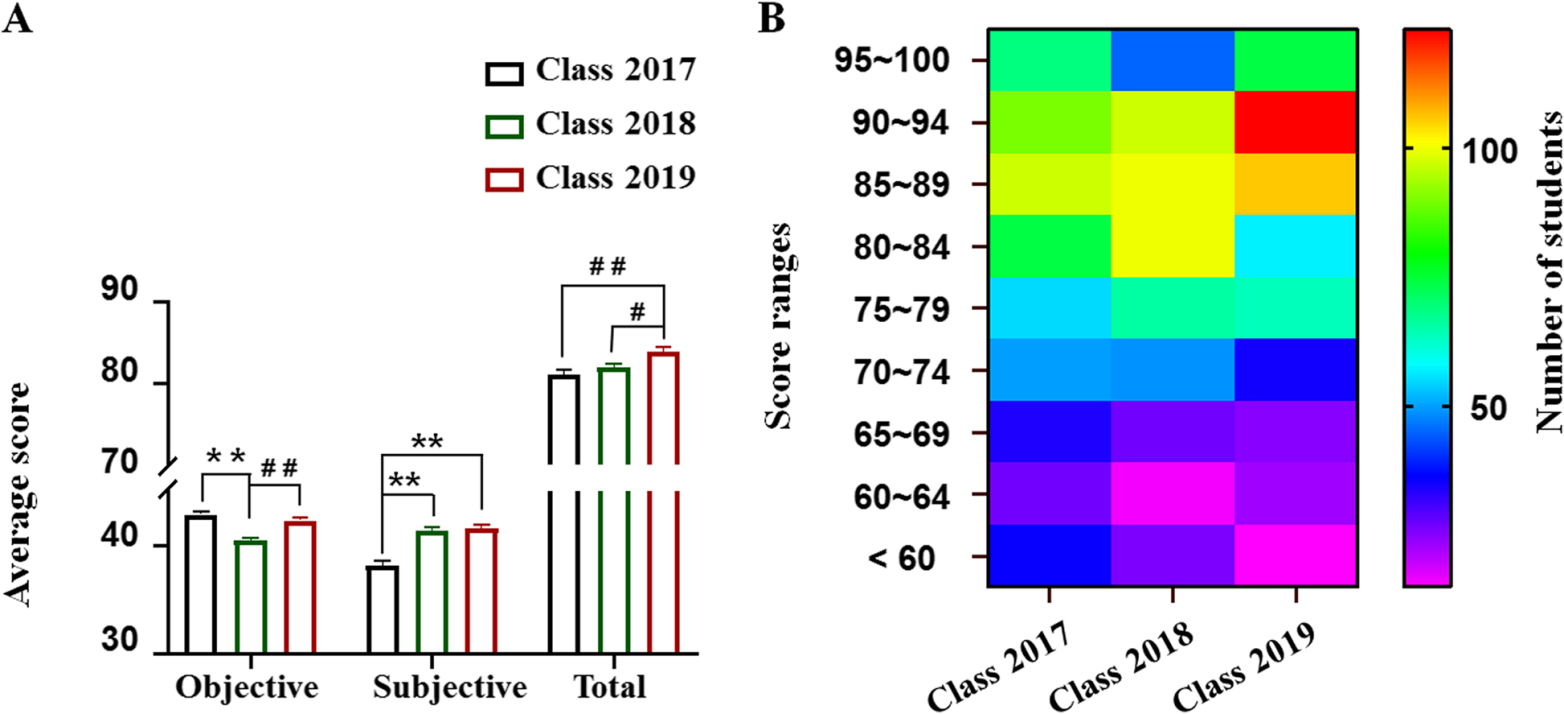
The difference and the distribution of students’ final examination scores among the three Classes. **A**. The comparison of average scores. **B**. The distribution of students’ scores. ** *P* < 0.01, compared to Class 2017; # *P* < 0.05, ## *P* < 0.01 compared to Class 2019

To evaluate the effectiveness of HBOPPPS, the scores of objective and subjective questions and the total scores of the final examination from Class 2017, 2018 and 2019 were compared. There was a significant increase in the total score of Class 2019 (83.9 ± 0.5) compared to Class 2017 (81.1 ± 0.6, *P* < 0.01) and Class 2018 (82.0 ± 0.5, *P* < 0.05) evaluated by ANOVA and Dunnett T3 test (Fig. 2A). Moreover, the average score of subjective questions was significantly higher in Class 2019 than that of Class 2017 (38.2 ± 0.4 in Class 2017 and 41.6 ± 0.3 in Class 2019, *P* < 0.01 by ANOVA and Dunnett T3 test).

It is worth noting that Class 2018 also had higher subjective question scores than Class 2017 (41.4 ± 0.3, *P* < 0.01 by ANOVA and Dunnett T3 test, Fig. 2A). However, the objective score of multi-choice and single answer questions earned by Class 2018 (40.5 ± 0.2) was significantly lower than that of the Class 2017 (42.9 ± 0.2, *P* < 0.01) and Class 2019 (42.3 ± 5.3, *P* < 0.01 by ANOVA and Bonferroni test, Fig. 2A).

Further analyzing the distribution of students’ number in different score sections revealed that the rate of excellence (scores between 90 and 100) of Class 2019 (197, 38.0%; *P* < 0.01 by chi-square test) was significantly higher than that of Class 2017 (159, 29.9%) and Class 2018 (142, 27.0%). Correspondingly, the rates of poor or failure (scores lower than 60) of Class 2019 (15, 2.9%; *P* < 0.05 by chi-square test) was significantly lower than that of Class 2017 (36, 6.8%) and Class 2018 (26, 4.9%, Fig. 2B).

**Fig. 3:**
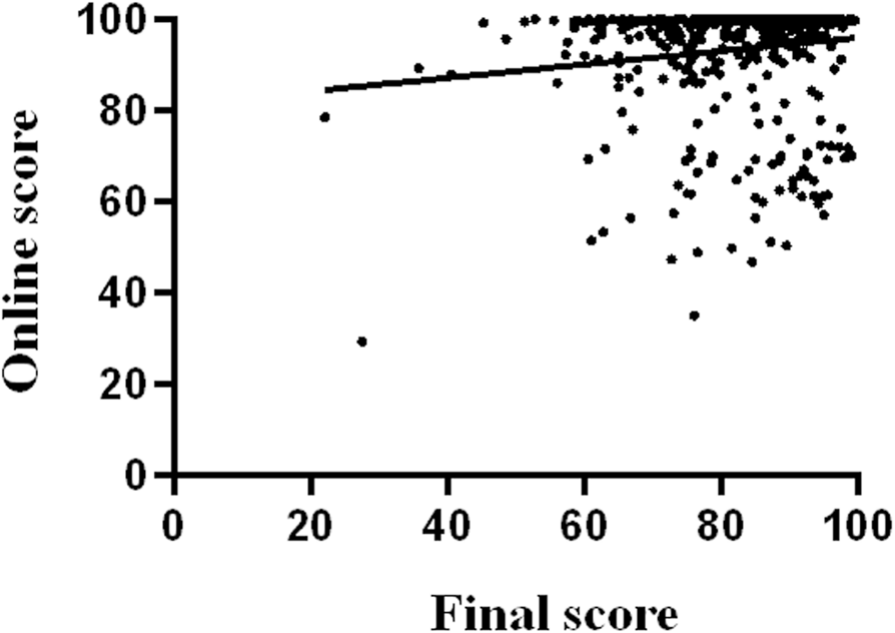
Correlation between the final examination score and the online score

With the assistance of the Mobile App, we could also give students an online score of Class 2019 automatically as an evaluation of students’ learning initiatives. The average online score of the four parts were as the follows: micro-lecture video watching (26.9 ± 0.37 points), homework for each chapter (38.1 ± 0.16 points), test (9.0 ± 0.08 points) and the attendance (19.8 ± 0.03 points), respectively. Analyzing the online score and its correlation with the final score revealed that there was a weak but significantly positive correlation between the online score and the final score (*r* = 0.14, *P* < 0.001 by Pearson correlation, Fig. 3).

### Students’ evaluation

**Fig. 4:**
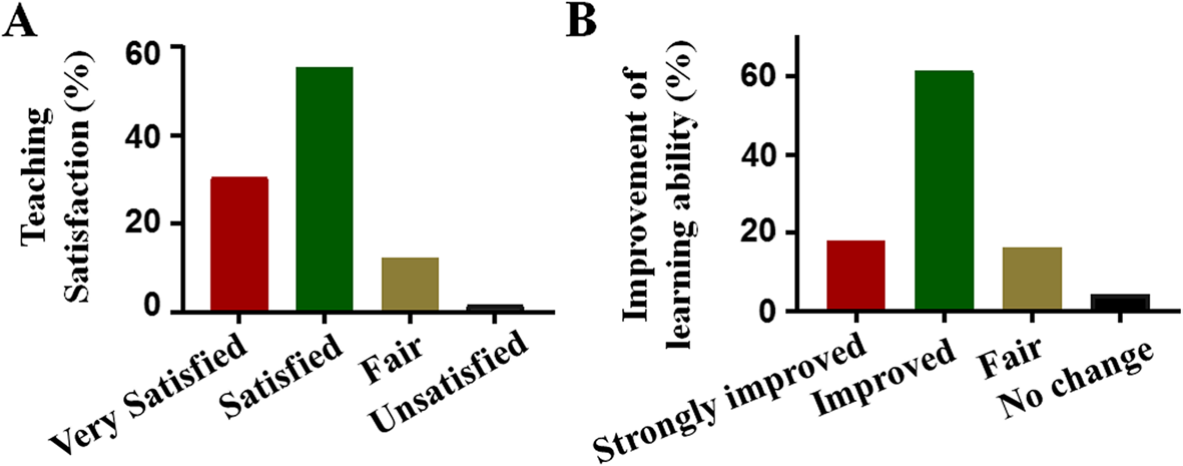
Percentage of students’ evaluation about HBOPPPS. **A**. Survey of students’ satisfaction. **B**. Survey of the improvement of students’ learning ability

Nowadays, with the development of quality education, the examination score is not the only criteria for assessing the quality of students. Learning ability is a very important criterion of assessment in the quality education. Thus, a questionnaire was performed in Class 2019 after the completion of whole Physiology courses to evaluate this HBOPPPS method. As shown in Fig. 4A, HBOPPPS satisfied 86.2% students and did not satisfy 1.6% of them because of several reasons that were analyzed later. There were also 79.4% of the students who perceived that HBOPPPS improved their learning ability. There were only 4.3% of the students who thought that HBOPPPS didn’t help very much and 16.2% of the students who did not give a clear answer (Fig. 4B).

### Advantages and disadvantages

**Fig. 5:**
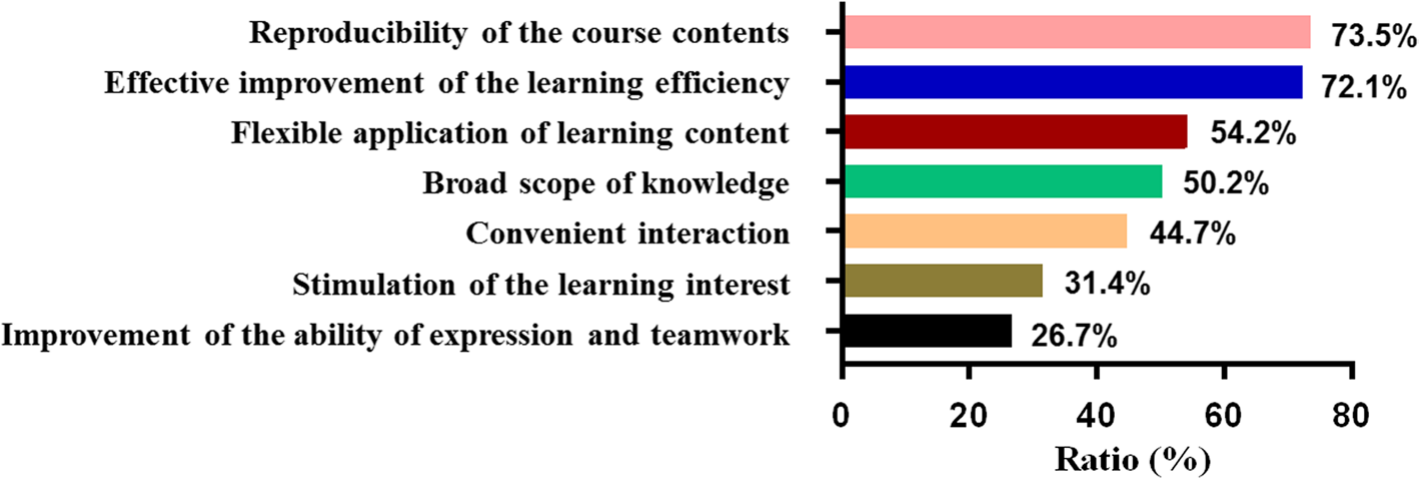
The advantages of HBOPPPS

Any teaching model has its own advantages and disadvantages. Our survey revealed that students perceived the following advantages of HBOPPPS. The two dominant reasons were conducive to reproduce the learning content after the lecture (73.5%) and effectively improved the learning efficiency (72.1%). In addition, ~ 50% of the students thought that HBOPPPS was flexible to apply the learning content (54.2%) and could broaden their knowledge (50.2%) (Fig. 5).

**Fig. 6:**
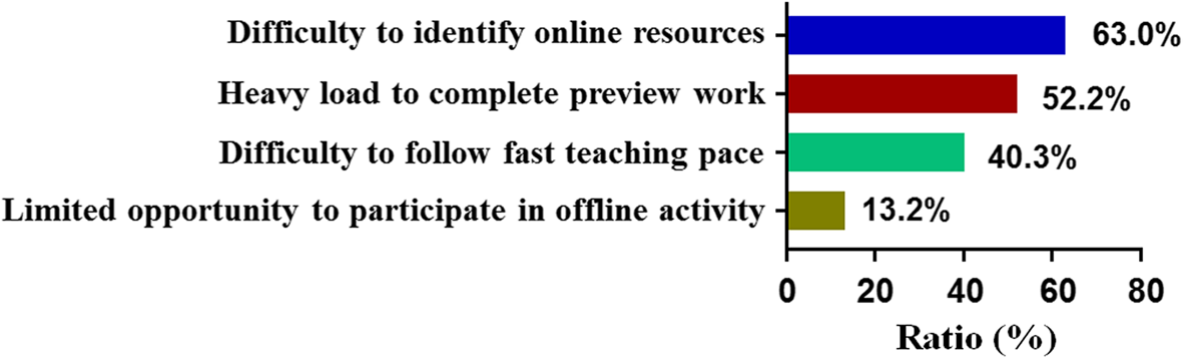
The disadvantages of HBOPPPS

By contrast, some disadvantages of this new teaching model were also perceived by the students. In online searching answers to discussion questions and expanding their knowledge, it was difficult to distinguish whether the resources that they found were suitable or not (63.0%), the preview task was heavy to accomplish (52.2%) and the teaching pace was too fast for some students to follow (40.3%) (Fig. 6).

### Students’ choice

**Fig. 7:**
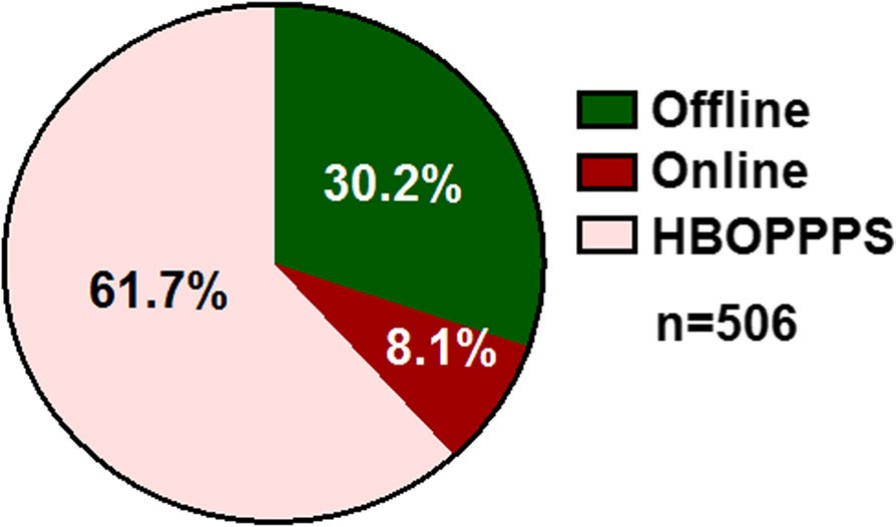
Students’ choices about teaching method in the future

At the end of the questionnaire, we asked students about their teaching model preference in Class 2019. Among 506 respondents, 61.7% of them showed a preference to the HBOPPPS (Fig. 7) while 30.2% still preferred to receive the traditional offline teaching which was used over the whole process before college. In addition, a small portion of the students (8.1%) preferred the online only teaching model.

## Discussion

The present study reveals that the HBOPPPS teaching model can significantly enhance the final score and the learning ability of students. This effectiveness is because HBOPPPS model takes full advantages of modified BOPPPS and the convenience of internet teaching and enhances learning initiative of students.

### Online teaching and modified BOPPPS

E-learning can be broadly defined as any type of educational media delivered through the internet [18]. Although it emerged in the middle of 1990s, the online course was not well established in China until 2020 when COVID-19 pandemic occurred [19]. Classically, web-based learning, such as video lectures and Massive Open Online Course [3–5], is recognized to help expand the scope of knowledge on the subject taught in classroom. However, online resources of learning themselves are not helpful for resolving individual difficult problems or stimulating students’ enthusiasm for learning, do not match the course objectives tightly and cannot flexibly link different sets of knowledge directly. Thus, it only serves as supplements for offline lecture for a long time. To resolve these problems, we provided course-bound micro-video lectures and online instructions/interactions for students to participate in the online teaching through the Mobile App. It can also calculate students’ activities automatically and provide timely feed-forward and feedback communications between teachers and students. Thus, the Mobile App becomes a powerful tool in establishing this HBOPPPS model.

In the HBOPPPS, TESOT contents and online teaching were partially incorporated into the BOPPPS-based teaching process, which aimed at compensating the insufficiency of classical BOPPPS. The six elements of BOPPPS [13] have their unique functions in enhancing learning efficiency. At first, bridge-in at class beginning draw the attention of students to the course (contents). Second, students can learn the aims of the course and have a general understanding of the content by emphasizing them before and at the beginning of lectures. Third, the pre-assessment is a challenge for students and guides their attention to the main lecture. Fourth, the modified BOPPPS emphasizes a participatory learning which puts stress on a student-centered approach and can inspire the initiative of students. Fifth, the post-assessment would let the teacher know how well the students have mastered the content and what they need to be taught immediately. Finally, a summary is necessary that can give the students a mind mapping of the course content and in turn strength their memory. Theoretically, these modifications make the HBOPPPS a more efficient teaching model.

### Evaluation of the effectiveness of the HBOPPPS

In China, almost all of the students of different majors take a final examination at the end of a semester while “formative assessment” that is based on multiple exams throughout the course is also taken by some institutes. In our study, the final score means one final exam at the end of a semester. This is because it can reflect the accumulating ability and knowledge of students’ learning. In addition, the different evaluation methods of non-final exam between different years also made the combination of scores from multiple exams unacceptable. In the final summative assessment, we also combined the online score that was taken as the index of students’ engaging in the course. The two scores together constitute a better index to reflect students’ knowledge and ability. Therefore, we used this final summative assessment but not the formative assessment to evaluate the effectiveness of the HBOPPPS. The better outcome of applying the HBOPPPS supports that in the era of internet plus, the HBOPPPS becomes necessary in achieving a better teaching outcome than either the online or offline teachings.

### Hybrid teaching method

Pure online teaching simulating offline teaching in BOPPPS model can partially resolve the limitation of classical web-based learning since it has clear goals and allows interactions between teacher and students directly. However, a 5-year study showed that the full online course have higher failure rate than the face-to-face offline course [20]. Our initial trial applied the immature HBOPPPS teaching model in Class 2018 by providing students with full lecture in BOPPPS model plus micro-video lecture online. The 80 home-made micro-lecture videos posted on the Mobile App allow the students to get a general idea about the ongoing lecture. Unexpectedly, this effort did not yield ideal results in their answering objective questions while some improvement in resolving subjective questions appeared. This is likely due to its lack of detailed contents required for answering the objective question. In addition, the structure of the classical BOPPPS model provided no much help while the introduction of micro-video deviated students’ attention away from the details of course contents. These facts led us to design the present HBOPPPS model that contains both the merits of online and offline teachings.

The results of our final summative assessment reveal that this hybrid teaching model can activate students’ interest, help them perform self-study and extend the depth of what they learned as previously reported [21]. In addition, students really appreciate their interactions with teachers given by the offline course and the possibility to perform small group work in an online environment. Correspondingly, they are mostly in favor of the hybrid course, a finding consistent with the reports from other groups [22, 23].

### Factors contributing to the effectiveness of HBOPPPS

The HBOPPPS group of Class 2019 had significantly better results than that of Class 2017 and 2018 for teaching, as measured by the final exam scores. Besides, Class 2019 also had higher rate of excellence and lower rate of failure, which indicate that the HBOPPPS had effectively improved students’ learning efficiency and outcomes. This finding was consistent with other reports that revealed the higher effectiveness of the hybrid teaching than traditional methods [24, 25].

Analyzing the reason of students’ improvement revealed a positive relationship of the final exam scores with the online score from the App’s calculation, which could reflect students’ activity on learning Physiology. Both the HBOPPPS offline and online teachings were designed to enhance students’ interests, activities and learning ability and thus, made them think and study independently and creatively. Students’ feedback showed that the advantages of HBOPPPS include the convenience to reproduce the course content and effective improvement of students’ learning efficiency. In addition, timely-feedback interactions between the teacher and students were also attributable to the better outcomes of HBOPPPS model.

Notably, the home-made micro-lecture videos are very popular among students; combining with the online guideline, they become an effective venue for the students to master the key knowledge in the lecture. This finding is consistent with other studies showed that there is a positive correlation between using micro-lectures and the final scores [26, 27]. The weak correlation coefficient between them is likely because the equally excellent performance of online activities of the students, a feature different from that of international medical students who have more liberal styles of learning [16].

Through analysis of the questionnaire, we could see that most of the students satisfied or very satisfied with this teaching model since the HBOPPPS did improve their learning ability. In Class 2019, 61.7% of students expressed their preference for using this hybrid teaching method in future studies. This finding gives us the confidence in performing the HBOPPPS in teaching of Physiology in future.

### Limitations and further improvements

Obviously, the HBOPPPS model largely resolves the limitations of classical online teaching model, compensates the space and time limitations of offline teaching, broadens students’ knowledge and enforces teacher-student interactions, and therefore has better teaching effectiveness. However, some disadvantages are also remarkable. Most of the students thought it was difficult to distinguish whether or not the online resources were suitable for discussion. This is a common problem for all of the people surfing the web in modern times since everyone can express their own ideas on the internet, high or low quality, and that need students to distinguish these messages carefully. Thus, looking for answers online clearly increases the burden of their study. Another concern of the students is the course burden that 30% of the students didn’t like this hybrid teaching because it was a heavy load to accomplish the preview task and course assignments. This is likely because Chinese students are used to traditional “filled-in” offline teaching, in which they just need to listen to and then remember the course. By contrast, this HBOPPPS model requires them to learn by themselves first and that could give them a kind of pressure. In our view, a pressure doesn’t mean bad since it can also be a driving force to promote college students’ ability of self-learning. About this issue, there was a clear instruction from the Chinese Ministry of Education that higher education needs to improve the degree of academic challenge, increases the difficulty of the course content and then increases the burden of students appropriately to eliminate “water courses” and create “golden courses”. Thus, teaching students how to adapt for this type of pressure will be a challenge for the teachers.

In addition, the improvement of students’ ability to answer multi-choice and single answer questions is not significant compared to the classical BOPPPS model. This seems because of that the HBOPPPS has not focused on keywords and definitions of individual facts while stressing on the analytical ability of the students due to the time limitation. Thus, these issues should be considered in composing the teaching materials. Lastly, the minor difference in the components of HBOPPPS among different teachers could also be a contributor, such as an option for interpreting the micro-video lecture or using pre-assessment with the quiz following the bridge-in, and students/teacher-led summary in the lecture, etc.

To make BOPPPS more effective, we have also incorporated some contents of the TESOT teaching model into the HBOPPPS. TESOT was created based on our identification of the learning obstacles and teaching style preferences of international medical students [17], which serve as a critical reference for designing a teaching modality to resolve these problems by using the components of active-learning classrooms [28] and online guidance [16]. In the present study, we also tried to incorporate some components of TESOT into the BOPPPS model including quizzes before and after the main lecture and partial students-led summary. However, the learning obstacles that revealed in the present and previous studies [17] were not fully considered, such as the course connections and limitations of self-studying time.

To further improve the efficiency of teaching Physiology, it is necessary to refine the structures and contents of HBOPPPS model while reducing students’ burden of self-learning. This effort, for example, can include putting stress on the interconnections between course/lectures by adjusting the contents of micro-video lectures and quizzes at the beginning of lecture (instead of simply generating a sound or showing a picture), making the online instructions clearer (assigning 1–2 associated chapter or short essays from a specific text book or online resources to read while viewing the micro-lecture video), and strengthening on-site digestion of course contents by fully applying students-led summary in the classroom and avoiding an open end of the online discussion/mind mapping. To reduce the general pressure in medical education, the institution may coordinate the teaching contents of associated academic disciplines by executing an integrative education strategy and thus make the “heavy load” in Physiological education be a relief of students’ learning the course of other disciplines.

## Conclusions

In the post-pandemic era of COVID-19, online teaching is a necessary portion of modern education. The HBOPPPS is the direction of future teaching since this reproducible and flexible approach of teaching during the COVID-19 pandemic is likely more effective than classical BOPPPS or its simple combination with online teaching although further studies and application in other courses are needed.

## Supplementary Information

**Additional file 1: Supplemental Table 1.** Arrangement of Physiology Course.

**Additional file 2: Supplemental Table2.** Course structures of the HBOPPPS.

**Additional file 3: Supplemental Table 3.** Examples of before and after lecture activities.

**Additional file 4: Supplemental Figure 1.** An example of mind map made after lecture. A, an original mind map made by a Chinese student; B, a translated version of mind map based on A.

**Additional file 5: Supplemental Table 4.** Exemplary contents of the final examination of Physiology course.

**Additional file 6: Supplemental Table 5.** Example of questionnaire.

**Additional file 7.**

## Data Availability

All data generated or analysed during this study are included in this published article and its supplementary information file-Raw data of students’ scores.

## Abbreviations

BOPPPS: A teaching method composed of six elements including bridge-in, learning objectives and outcomes, pre-assessment, participatory learning, post-assessment and summary
HBOPPPS: A hybrid teaching method incorporating online teaching into BOPPPS components
TESOT: A teaching modality enriched with components of student-centered approach and online interactions targeting the learning obstacles in global medical education
