# Supplementary material for: Assessment of the effectiveness of BOPPPS-based hybrid teaching model in physiology education
Source: BMC Med Educ. 2022 Mar 30;22:217. doi: 10.1186/s12909-022-03269-y (PMC8966603; doi:10.1186/s12909-022-03269-y)
Supplement: Supplementary file 1 — Additional file 1: Supplemental Table 1. Arrangement of Physiology Course. [file 12909_2022_3269_MOESM1_ESM.docx]

**Assessment of the effectiveness of BOPPPS-based** **hybrid teaching model in Physiology education**

Xiao-Yu Liu, Chunmei Lu, Hui Zhu, Xiaoran Wang, Shuwei Jia, Ying Zhang, Haixia Wen, and Yu-Feng Wang

Supplemental table 1. Arrangement of Physiology Course

| **Main contents** | **Sessions/45min** | **Teachers** |
| --- | --- | --- |
| Introduction | 2 | Teacher A |
| Cells and its functions | 8 | Teacher A |
| Blood | 4 | Teacher B |
| Circulation | 14 | Teacher B |
| Respiration | 6 | Teacher C |
| Digestion and absorption | 6 | Teacher C |
| Energy metabolism and body temperature | 4 | Teacher D |
| Urine production and excretion | 8 | Teacher D |
| Function of the sensory organs | 6 | Teacher E |
| Function of the nervous system | 12 | Teacher E |
| Endocrine | 8 | Teacher F |
| Reproduction | 2 | Teacher F |
