# Supplementary material for: Assessment of the effectiveness of BOPPPS-based hybrid teaching model in physiology education
Source: BMC Med Educ. 2022 Mar 30;22:217. doi: 10.1186/s12909-022-03269-y (PMC8966603; doi:10.1186/s12909-022-03269-y)
Supplement: Supplementary file 2 — Additional file 2: Supplemental Table2. Course structures of the HBOPPPS. [file 12909_2022_3269_MOESM2_ESM.docx]

**Assessment of the effectiveness of BOPPPS-based** **hybrid teaching model in Physiology education**

Xiao-Yu Liu, Chunmei Lu, Hui Zhu, Xiaoran Wang, Shuwei Jia, Ying Zhang, Haixia Wen, and Yu-Feng Wang

Supplemental table 2. Course structures of the HBOPPPS

| **Main contents** | **Time** | **Way of presentation** | **Purpose** |
| --- | --- | --- | --- |
| Bridge in | Before lecture | Online discussions, expanded knowledge, science stories, micro-video lecture | Attract students’ attention |
| Activity in the classroom | 90 min |  |  |
| Check attendance | At the beginning of lecture | Online (Mobile App) | A composition of the students’ online score |
| Objective and expected outcomes | 2 min | Online and PPT shows course introduction and what students will gain from the course | Provide overview of the course |
| Pre-assessment | 3 min | One multiple choice-single answer questions (Mobile App) | Link background knowledge and provide hints for the ongoing lecture |
| Participatory learning | 75 min | PPT, lectures, handwriting on the blackboard, implication questions, group discussion, dynamic pictures, videos, raise random questions, mobile phone shake to select a student randomly (Mobile App) | Attract students’ attention, activate the initiative of students, participatory learning, make sure students understood key points |
| Post- assessment | 5 min | 3~5 multiple choice-single answer questions (Mobile App) | Check teaching efficiency and remained problems |
| A students-led summary under the supervision of teacher | 5 min | Summarize the key contents based on blackboard writing with emphasis marks and associated figures in PPT | Activate the initiative of students, enhance students’ understanding, verify full understanding of the key contents |
| Mind map or topics | After lecture | Post online for student-led summary and discussion | Deepen understanding and memory, easy for review |
| Post-lecture interactions (mentoring) | After lecture | Online (posting questions, private chat with teachers, provide feedback information) | Strengthen relationships with students, resolve difficult issues, forming a positive feedback loop with students |
