# Supplementary material for: Assessment of the effectiveness of BOPPPS-based hybrid teaching model in physiology education
Source: BMC Med Educ. 2022 Mar 30;22:217. doi: 10.1186/s12909-022-03269-y (PMC8966603; doi:10.1186/s12909-022-03269-y)
Supplement: Supplementary file 3 — Additional file 3: Supplemental Table 3. Examples of before and after lecture activities. [file 12909_2022_3269_MOESM3_ESM.docx]

**Assessment of the effectiveness of BOPPPS-based** **hybrid teaching model in Physiology education**

Xiao-Yu Liu, Chunmei Lu, Hui Zhu, Xiaoran Wang, Shuwei Jia, Ying Zhang, Haixia Wen, and Yu-Feng Wang

Supplemental Table 3. Examples of before and after lecture activities.

| **Pre-Assessment** | **Post-Assessment** |
| --- | --- |
| **What stimuli are the smooth muscles of the digestive tract sensitive to？**  A. Temperature  B. Chemicals  C. Mechanical stretch  D. Cut  E. Electrical stimulation | **Which one determines the strength of gastrointestinal smooth muscle contraction?**  A. The frequency of the slow wave  B. The amplitude of the slow wave  C. The frequency of the action potentials  D. The amplitude of the action potential  E. The intrinsic properties of smooth muscle |
