# Supplementary figures and images for: Assessment of the effectiveness of BOPPPS-based hybrid teaching model in physiology education
Source: BMC Med Educ. 2022 Mar 30;22:217. doi: 10.1186/s12909-022-03269-y (PMC8966603; doi:10.1186/s12909-022-03269-y)

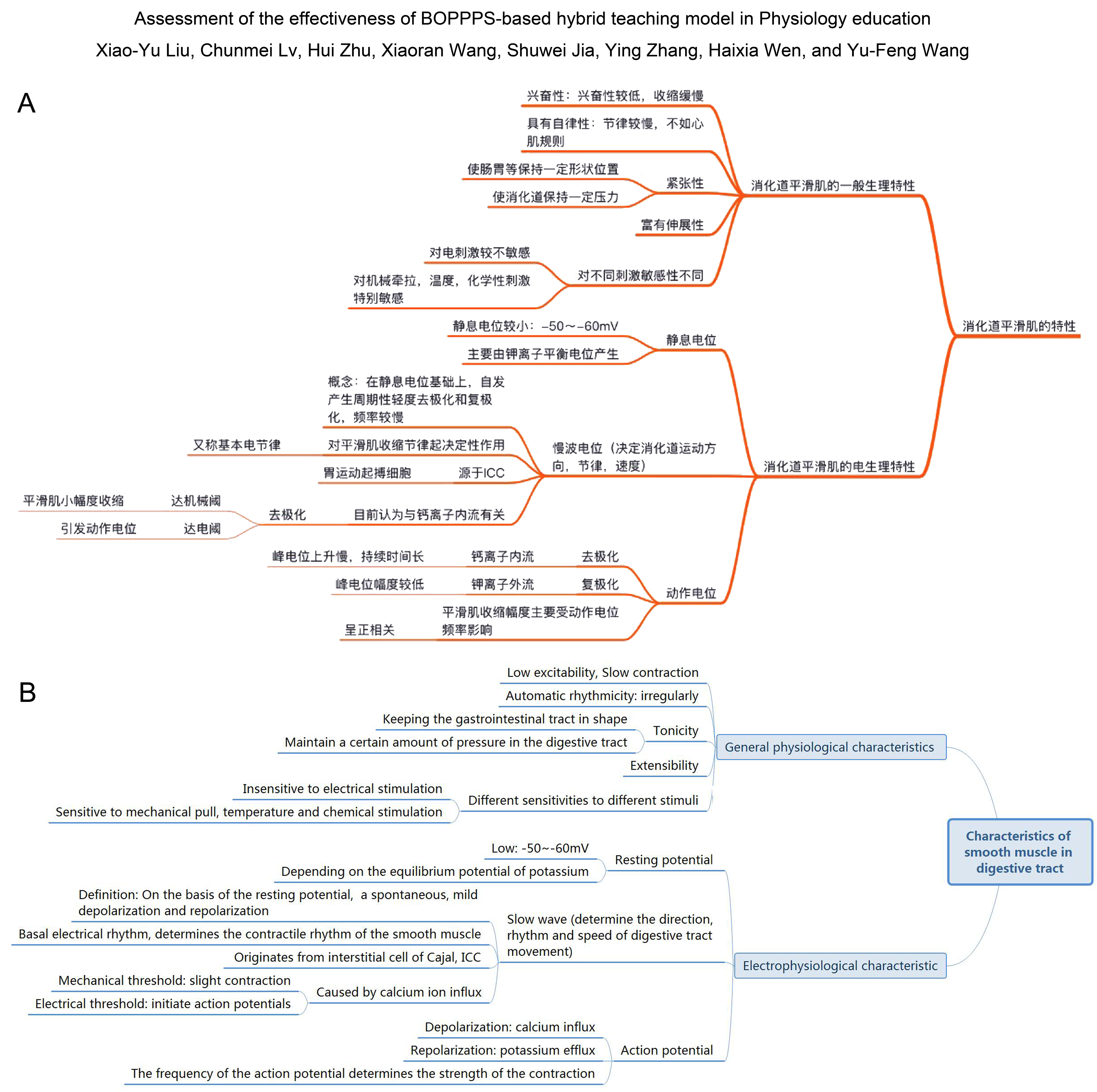

Supplement: Supplementary file 4 — Additional file 4: Supplemental Figure 1. An example of mind map made after lecture. A, an original mind map made by a Chinese student; B, a translated version of mind map based on A. [file 12909_2022_3269_MOESM4_ESM.tif]
