# Supplementary material for: Assessment of the effectiveness of BOPPPS-based hybrid teaching model in physiology education
Source: BMC Med Educ. 2022 Mar 30;22:217. doi: 10.1186/s12909-022-03269-y (PMC8966603; doi:10.1186/s12909-022-03269-y)
Supplement: Supplementary file 5 — Additional file 5: Supplemental Table 4. Exemplary contents of the final examination of Physiology course. [file 12909_2022_3269_MOESM5_ESM.docx]

**Assessment of the effectiveness of BOPPPS-based** **hybrid teaching model in Physiology education**

Xiao-Yu Liu, Chunmei Lu, Hui Zhu, Xiaoran Wang, Shuwei Jia, Ying Zhang, Haixia Wen, and Yu-Feng Wang

Supplemental Table 4. Exemplary contents of the final examination of Physiology course

| **1.** Multiple-choice and single answer questions (1 point for each question, 50 points in total)  1.1 The internal environment means: ( B )  A. Intracellular fluid  B. Extracellular fluid  C. Interstitial fluid  D. Plasma  E. Lymphatic fluid  ,,, ,,,  1.50 XXX |
| --- |
| **2.** Short answer questions (5 points for each question, 30 points in total)  2.1 What are the factors that affect arterial blood pressure?  **Answer**: Stroke Volume (1 point), Heart Rate (1 point), Peripheral Resistance (1 point), Function of windkessel vessels (1 point), Mean circulatory filling pressure (1 point).  ,,, ,,,  2.6 XXX |
| **3.** Essay questions (10 points for each question, 20 points in total).  3.1 Please describe the stages of action potential of ventricular myocytes and the main ion flows in each stage.  **Answer**: There are five stages of action potential process of ventricular myocyte, 1) Phase 0 (depolarization) (1 point), fast sodium channels open, sodium ions rapidly flow into the cell (1 point)); Phase 1 (initial repolarization) (1 point), fast sodium channels close and the potassium ions leave the cell through opening potassium channels (1 point); Phase 2 (plateau) (1 point), calcium channels open and potassium channels close. Increased calcium ion flowing into the cell and decreased potassium ion leaving the cell (1 point); Phase 3 (rapid repolarization) (1 point), calcium channels close and slow potassium channels open. Potassium ions exit the cell rapidly (1 point); Phase 4 (resting membrane potential) (1 point) averages about −90 millivolts. Ions return to normal levels (1 point).  3.2 XXX |
