# Supplementary material for: Assessment of the effectiveness of BOPPPS-based hybrid teaching model in physiology education
Source: BMC Med Educ. 2022 Mar 30;22:217. doi: 10.1186/s12909-022-03269-y (PMC8966603; doi:10.1186/s12909-022-03269-y)
Supplement: Supplementary file 6 — Additional file 6: Supplemental Table 5. Example of questionnaire. [file 12909_2022_3269_MOESM6_ESM.docx]

**Assessment of the effectiveness of BOPPPS-based** **hybrid teaching model in Physiology education**

Xiao-Yu Liu, Chunmei Lu, Hui Zhu, Xiaoran Wang, Shuwei Jia, Ying Zhang, Haixia Wen, and Yu-Feng Wang

Supplemental Table 5. Example of questionnaire.

| **Questions** | **Answers** |
| --- | --- |
| **Do you like Physiology?**  Very much  Like  Fair  No  **Are you satisfied with the Physiology course?**  Very much  Satisfied  Fair  Unsatisfied  **Does the online course in physiology improve your learning ability?**  Strongly improved  Improved  Fair  No change  **What do you think are the advantages of the Physiology course?**  Reproducibility of the course contents  Effectively improvement of the learning efficiency  Flexible application of learning content  Broad scope of knowledge  Convenient interaction  Stimulation of the learning interest  Improvement of the ability of expression and teamwork  **What do you think are the disadvantages of the Physiology course?**  Difficulty to identify online resources  Heavy load to complete preview work  Difficulty to follow fast teaching pace  Limited opportunity to participate in offline activity  **Which method of teaching modality do you prefer?**  Online teaching  Offline teaching  Hybrid teaching | 120 (23.72%)  303 (59.88%)  81 (16.01%)  2 (0.4%)  155 (30.63%)  281 (55.53%)  62 (12.25%)  8 (1.58%)  91 (17.98%)  311 (61.46%)  82 (16.21%)  22 (4.35%)  372 (73.52%)  365 (72.13%)  274 (54.15%)  254 (50.2%)  226 (44.66%)  159 (31.42%)  135 (26.68%)  319 (63.04%)  264 (52.17%)  204 (40.32%)  67 (13.24%)  41 (8.1%)  153 (30.24%)  312 (61.66%) |
